# Novel segmentally deployable self-expandable metallic stent in malignant colorectal obstruction

**DOI:** 10.1055/a-2695-0679

**Published:** 2025-09-15

**Authors:** Takashi Murakami, Tomonori Yamauchi, Eiji Kamba, Sho Takahashi, Yusuke Takasaki, Akihito Nagahara, Hiroyuki Isayama

**Affiliations:** 1 Department of Gastroenterology, Juntendo University, Postgraduate School of Medicine, Tokyo, Japan; 2 Department of Pathophysiological Research and Therapeutics for Gastrointestinal Disease, Juntendo University Faculty of Medicine, Tokyo, Japan

## Introduction

Deployment of segmentally deployable self-expandable metallic stents in two cases of malignant colorectal obstruction.Video 1

**Fig. 1:**
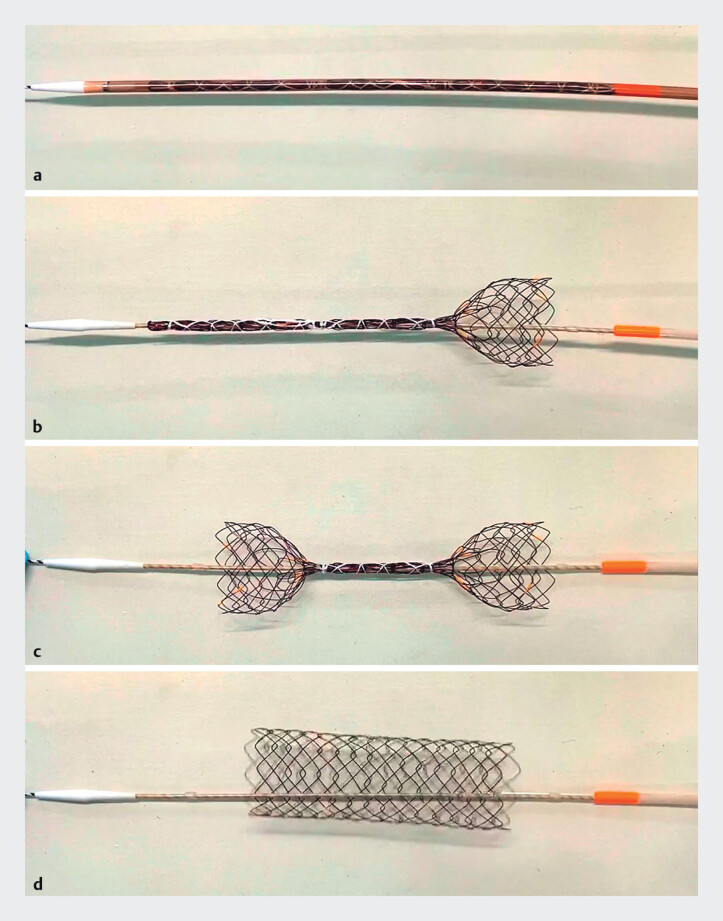
Demonstration of deployment of the segmental-release self-expandable metallic stent (SEMS), Bactrian SR colonic stent (SB-Kawasumi Laboratories Inc., Kanagawa, Japan), in vitro. **a** SEMS before deployment. The SEMS was deployed in the following order: **b** anal, **c** oral, and **d** central segments. These segments can be released at any order of magnitude.

We report two successful cases of colonic stenting using a newly developed self-expandable metallic stent (SEMS) with suture-based segmental-release deployment system (SR), the Bactrian SR colonic stent (SB-Kawasumi Laboratories Inc., Kanagawa, Japan). This novel uncovered SEMS is made from nickel-titanium features hook-and-cross braided design that provides low axial and sufficient radial forces 1 . The delivery system has a three-layer design comprising an outer sheath, suture-based deployment system, and inner catheter. This system has three independently deployable segments — oral, central, and anal — whose deployment order can be selected ( Fig. 1 , Video 1 ). Compared with the conventional one-step outer sheath pulling-back deployment, the deployment order from oral/anal deployment at the center may be more effective for precise placement 2 .

## Case report

Case 1 was an 82-year-old man undergoing chemotherapy for transverse colon cancer who presented with vomiting and abdominal distension. Colonoscopy revealed a near-circumferential obstructive tumour with a 9-cm stricture (ColoRectal Obstruction Scoring System (CROSS) 3 score of 1). A 22 mm × 12 cm SEMS was deployed in three steps — oral, anal, and central — with accurate adjustments under fluoroscopic guidance.

**Fig. 2:**
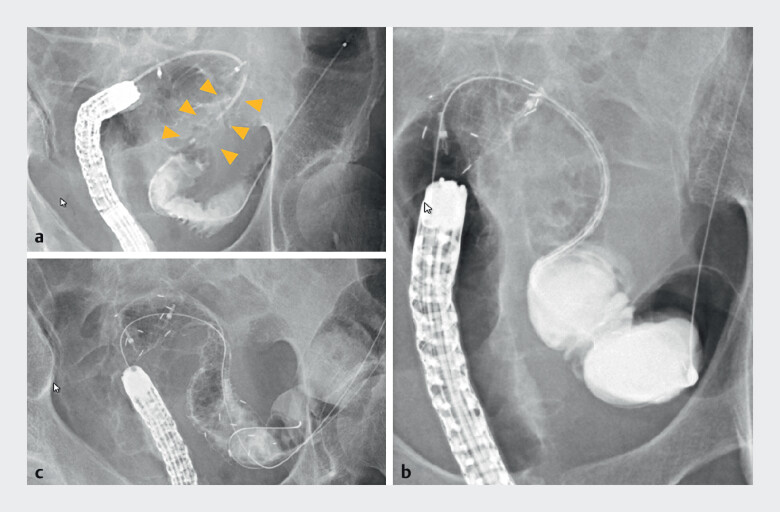
Stepwise deployment of a segmental-release SEMS in Case 2. **a** Fluoroscopic image showing a 4-cm stricture at the sigmoid colon flexure. **b** Initial deployment of the oral and anal segments of the stent using the segmental-release system. **c** Final deployment of the central segment after adjusting the stent position to ensure the stricture is centred within the stent.

Case 2 was an 88-year-old man with appetite loss and vomiting who was diagnosed with sigmoid colon cancer and liver metastases (CROSS 3 score of 1). Colonoscopy confirmed a type 2 tumor causing a 4-cm stricture. Although endoscopic access was unstable owing to the location of the lesion, a 22 mm × 12 cm SEMS with suture-based segmental release was successfully deployed ( Fig. 2 ).

In both cases, segmental release was effective and placing the SEMS at the expected portion precisely was possible. Patients resumed oral intake on postoperative Day 2 and showed no adverse events for 2 months after stent placement.

## Conclusions

These cases highlight the utility of segmentally deployable SEMS in managing malignant colorectal obstruction, especially in unstable or angulated segments, where precise positioning is essential to avoid complications, such as perforation.

## References

[1] Isayama H Nakai Y Toyokawa Y Measurement of radial and axial forces of biliary self-expandable metallic stents Gastrointest Endosc 2009 70 37 44 10.1016/j.gie.2008.09.032 19249766

[2] van Hooft JE Veld JV Arnold D Self-expandable metal stents for obstructing colonic and extracolonic cancer: European Society of Gastrointestinal Endoscopy (ESGE) Guideline - Update 2020 Endoscopy 2020 52 389 407 10.1055/a-1140-3017 32259849

[3] Saida Y Current status of colonic stent for obstructive colorectal cancer in Japan; a review of the literature J Anus Rectum Colon 2019 3 99 105 10.23922/jarc.2019-009 31583324 PMC6774736

